# Differential Impact of Massachusetts, Canadian 4/91, and California (Cal) 1737 Genotypes of Infectious Bronchitis Virus Infection on Lymphoid Organs of Chickens

**DOI:** 10.3390/v16030326

**Published:** 2024-02-21

**Authors:** Reham M. Abd-Elsalam, Shahnas M. Najimudeen, Motamed E. Mahmoud, Mohamed S. H. Hassan, Rodrigo A. Gallardo, Mohamed Faizal Abdul-Careem

**Affiliations:** 1 Faculty of Veterinary Medicine, University of Calgary, Health Research Innovation Center 2C53, 3330 Hospital Drive NW, Calgary, AB T2N 4N1, Canada; reham.abdelsalam1@ucalgary.ca (R.M.A.-E.); fathimashahnas.moham@ucalgary.ca (S.M.N.); motamed.ali@ucalgary.ca (M.E.M.); msh.hassan@ucalgary.ca (M.S.H.H.); 2 Department of Pathology, Faculty of Veterinary Medicine, Cairo University, Giza 12211, Egypt; 3 Department of Animal Husbandry, Faculty of Veterinary Medicine, Sohag University, Sohag 82524, Egypt; 4 Department of Avian and Rabbit Medicine, Faculty of Veterinary Medicine, Assiut University, Assiut 71515, Egypt; 5 Department of Population Health and Reproduction, School of Veterinary Medicine, University of California Davis, 1089 Veterinary Medicine Drive, 4008 VM3B, Davis, CA 95616, USA; ragallardo@ucdavis.edu

**Keywords:** infectious bronchitis virus, Massachusetts genotype, Canadian 4/91 genotype, California 1737 genotype, harderian gland, bursa of Fabricius, cecal tonsils, spleen, chicken

## Abstract

Infectious bronchitis virus (IBV) induces severe economic losses in chicken farms due to the emergence of new variants leading to vaccine breaks. The studied IBV strains belong to Massachusetts (Mass), Canadian 4/91, and California (Cal) 1737 genotypes that are prevalent globally. This study was designed to compare the impact of these three IBV genotypes on primary and secondary lymphoid organs. For this purpose, one-week-old specific pathogen-free chickens were inoculated with Mass, Canadian 4/91, or Cal 1737 IBV variants, keeping a mock-infected control. We examined the IBV replication in primary and secondary lymphoid organs. The molecular, histopathological, and immunohistochemical examinations revealed significant differences in lesion scores and viral distribution in these immune organs. In addition, we observed B-cell depletion in the bursa of Fabricius and the spleen with a significant elevation of T cells in these organs. Further studies are required to determine the functional consequences of IBV replication in lymphoid organs.

## 1. Introduction

Infectious bronchitis (IB) is a serious and economically significant disease in chickens, caused by a virus belonging to the Coronaviridae family, called infectious bronchitis virus (IBV). Since the disease was originally recognized in a North Dakota poultry flock in 1931 [1], it has become one of the most devasting diseases for poultry flocks worldwide. The ongoing emergence of new antigenic variants due to genomic mutations and recombination [2] has made it extremely difficult to prevent and control IB with vaccinations [3]. These IBV variants may differ in pathogenicity, induced cellular response, and tissue tropism [4]. They can escape vaccine-elicited immune responses [5], leading to ineffective IB prevention programs.

The Massachusetts (Mass) genotype of IBV was initially recognized in the United States [6,7,8]. Since then, Mass-type IBV strains have been isolated in Europe, Asia, Africa, Australia, and South America [9,10,11]. The Mass strains of IBV have been prevalent in Canada [12,13], causing sporadic outbreaks in layer flocks of variable ages and impacting egg production in Western Canada [14,15].

The 4/91 IBV genotype has become a pivotal variant worldwide because of its rapid spread and reported vaccine breaks [16,17]. This genotype has a broad tissue tropism including the respiratory tract, kidney, gastrointestinal tract, and immune organs [17,18,19]. It caused a major dilemma in the poultry industry in Canada due to its wide tissue tropism and lack of cross-protection from available vaccines [20]. The Canadian 4/91 IBV was first identified in the middle of 2011 when 4/91 vaccinations were not being used [13]. From 2013 to 2017, serious production and respiratory issues in chicken flocks due to the Canadian 4/91 IBV strain were reported [13,17,21].

In California, in the 1990s, different IBV isolates capable of evading vaccine protection and causing outbreaks were classified as California variants [22,23]. IBV California (Cal) 1737 was initially identified in 2003–2004 and isolated from the respiratory tract, kidneys, and cecal tonsils (CT) of 6-wk-old chickens [24]. The pathology of Cal 1737 is generally characterized by respiratory illness in broilers aged 32 to 46 days and lymphoplasmacytic nephritis in chickens aged 4 to 6 weeks [25]. Consequently, Cal 1737 and Cal 99 variants have been considered the most predominant genotypes in North America [26,27]. Cal 1737 genotype of IBV was isolated from backyard chickens and commercial poultry in California State, according to a recent study [28].

Primary immune organs such as the thymus and bursa of Fabricius (BF) are responsible for the generation of T and B cells, respectively [29]. Meanwhile, the secondary immune organs; Harderian gland (HG), spleen, and CT aid in T- and B-cell maturation [30] and the generation of immune responses against diverse antigens. Targeting of these immune organs by IBV has been confirmed by several studies [19,31,32,33,34,35]. Furthermore, the replication of IBV in HG, BF, and CT with subsequent induced pathological lesions may have a functional impact on the bird’s immune response [32,33,34]. 

Both humeral and cell-mediated immune responses can be triggered by IBV infection [36,37,38]. While antibody-mediated immune response to IBV is crucial, it is not sufficient to shield birds from IBV-induced respiratory and renal illnesses [36,39]. In contrast, cell-mediated immunity is critical for eliminating IBV and reducing viral shedding [40,41]. Previous studies have addressed the role of cytotoxic T cells in controlling IBV replication and spread during the initial stage of infection. Reduced viral genome loads in the lungs and kidneys of IBV-infected birds were correlated with increased cytotoxic T lymphocyte (CTL) activity in the spleen [8,36,42,43].

Although Canadian 4/91, Mass, and Cal 1737 genotypes of IBV were previously recognized and classified as respiratory, renal, and reproductive tract pathogens in chickens [13,17,26], data on their impact on the HG, thymus, spleen, BF, and CT of chickens are very limited. Therefore, in our study, we aimed to investigate the impact of Canadian 4/91, Mass, and Cal 1737 strains of IBV on histological changes, tissue tropism, viral replication, and immune cell numbers in these immune organs. These data provide touchstones in understanding the potential immune-suppressive effect of those IBV strains on chickens.

## 2. Materials and Methods

### 2.1. Virus and the Animals

Three different IBV strains, namely Canadian 4/91, Mass, and Cal 1737, designated as IBV/Ck/Can/17-038913 [17], 15AB-01 [15], and Cal 1737-04, respectively, were used in this experiment. The Cal 1737-04 IBV was isolated from tracheal swabs obtained from broiler chickens (25 days old) with respiratory manifestations in California, USA. The virus isolation and propagation were conducted in 9–11-days old specific pathogen-free (SPF) embryonated chicken eggs as previously described [14,44]. The titration of the isolated IBV strains was performed following the laboratory manual for the isolation and identification of avian pathogens from AAAP and Reed and Muench’s method [45,46].

For the experimental study, one-day-old specific pathogen-free (SPF) chickens were purchased from the Canadian Food Inspection Agency (CFIA), Ottawa. The chickens were housed at the Veterinary Science Research Station (VSRS) at the Spy Hill campus, University of Calgary, and they were allowed to adapt to this environment for six days with ad libitum feed and water. The ethical approval for this work was obtained from the Veterinary Science Animal Care Committee (HSACC) of the University of Calgary (Protocol number: AC19-0011).

### 2.2. Experimental Infection and Sample Collection

One-week-old SPF chickens (n = 48) were randomly divided into four groups (n = 12 per group) where three groups were infected with 100 µL of IBV Mass, 4/91, and Cal 1737 variants at a dose of 1 × 10^5^ egg infectious dose (EID) _50_/bird via the oculo-nasal route while maintaining a mock-infected control group. Following infection, the chickens were monitored daily for clinical signs and clinical manifestations, which were scored as described previously [47].

At 3 and 7 days post-infection (dpi), blood, oropharyngeal (OP), and cloacal (CL) swab samples were collected from a randomly selected birds (n = 6 per group). Then, the birds were anesthetized by over-inhalation of isoflurane and euthanized by cervical dislocation for postmortem examination and to collect HG, thymus, BF, CT, and spleen tissue samples. 

The collected swabs were stored in 1 mL aliquots of PBS, while tissue samples were stored in RNA Save^®^ at −80 °C (Biological Industries, Beit Haemek, Israel) for extraction of ribonucleic acid (RNA). In addition, for histopathology and immunohistochemistry, tissue samples were fixed in 10% neutral buffered formalin (VWR International, Edmonton, AB, Canada).

### 2.3. Techniques

#### 2.3.1. RNA Extraction and cDNA Synthesis

RNA extraction was performed using Trizol LS^®^ and Trizol reagents (Ambion, Invitrogen Canada Inc., Burlington, ON, Canada) for the swabs and tissues, respectively, according to the manufacturer’s procedures. A Nanodrop 1000 spectrophotometer (Thermo Scientific, Wilmington, DE, USA) was used to measure the purity and concentration of extracted RNA. Complimentary (c) DNA synthesis was performed following the manufacturer’s guidelines using RT random primers (high-capacity cDNA reverse transcriptase kit, Invitrogen Life Technologies, Carlsbad, CA, USA).

#### 2.3.2. IBV Genome Load Quantification by Quantitative (q)PCR Technique

IBV genome load quantification in cDNA samples was performed by qPCR technique, using the Fast SYBR^®^ Green master mix (Quntabio, Beverly, MA, USA) and CFX 96-c1000 Thermocycler (Bio-Rad Laboratories, Mississauga, ON, Canada) as previously described [48].

The thermal cycling conditions were as follows: (I) initial denaturation at 95 °C for 20 s, then 40 cycles of (II) denaturation at 95 °C for 3 s and (III) annealing at 60 °C for 30 s. The sequences of the primers used are as follows: IBV forward 5′GACGGAGGACCTGATGGTAA-3′ and reverse 5′CCCTTCTTCTGCTGATCCTG-3′ [48].

#### 2.3.3. Histopathological Examination

The HG, BF, thymus, spleen, and CT harvested from the experimental groups at 3 and 7 dpi and fixed in 10% neutral buffer formalin (VWR International, West Chester, PA, USA) were submitted to the Diagnostic Services Unit (DSU) of the University of Calgary, Faculty of Veterinary Medicine, to obtain hematoxylin and eosin (H&E) stained and unstained slides (positively charged) for immunohistochemistry assay. The histopathological lesion scoring of the HG, BF, thymus, spleen, and CT was performed according to the criteria (Table 1) established previously [48]. In brief, the scoring was performed as follows: normal (0); mild (1); moderate (2); severe (3) for each criterion, and then the total score was calculated for each organ.

#### 2.3.4. Immunohistochemistry Assays

The immunohistochemical staining for IBV antigens and T-cell and B-cell markers was performed following the methods described by [48,49]. Briefly, after deparaffinization in xylene and rehydration in alcohol, the endogenous peroxidase activity was blocked using a 3% H_2_O_2_ solution in methanol for 10 minutes (min). Heat antigenic retrieval was conducted by microwaving the tissue specimens with a 10 mM citrate buffer, pH 6.0 for 17 min at 850 V. The tissue specimens were incubated overnight in the fridge with one of the following antibodies: mouse primary anti-IBV nuclear protein antibody (Novus Biological, Bio-Techne, Toronto, ON, Canada) diluted 1:400, monoclonal mouse anti-human CD3 antibody (Abcam, Toronto, ON, Canada) diluted 1:100, and monoclonal mouse anti-chicken BU-1 antibody (Southern Biotech, Birmingham, AL, USA) diluted 1:200. The secondary antibody, goat anti-mouse IgG (H+L) (DK-2594, Vector Laboratories Inc., Newark, CA, USA), ABC peroxidase kit, and 3,3′-Diaminobenzidine (DAB) substrate solution (Vector Laboratories, Newark, CA, USA) were used to detect primary antibody binding. Finally, the tissue sections were counterstained using Hematoxylin (Vector Laboratories, Inc., Newark, CA, USA), cover-slipped, and mounted. All sections were examined under a microscope to detect the presence of the IBV antigen. The number of organs per each group that revealed positive immune staining against the IBV antigen was counted out of 6 (number of birds per group). Concerning BU-1- and CD3-stained slides, 5 different fields per tissue from each animal in the group were examined using a 200× magnification, photographed, and subsequently analyzed. To calculate the percentage of immune-positive cells, Image J analyzer software version 1.46a (National Institute of Health, Bethesda, MD, USA) was used.

### 2.4. Statistical Analysis

A two-way ANOVA followed by Tukey’s multiple comparison test was used to detect the differences in IBV genome loads obtained from OP swabs, CL swabs, and tissues, as well as the BU-1 and CD3 immune-positive cell % among four different experimental groups at 3 and 7 dpi. The histopathological lesion scores were compared and analyzed using Kruskal–Wallis’s test followed by Dunn’s multiple comparison. All the data analyses were conducted using GraphPad Prism 10.0.0 Software (San Diego, CA, USA).

## 3. Results

### 3.1. IBV Genome Loads in Swabs and Tissues Samples

**Figure 1:**
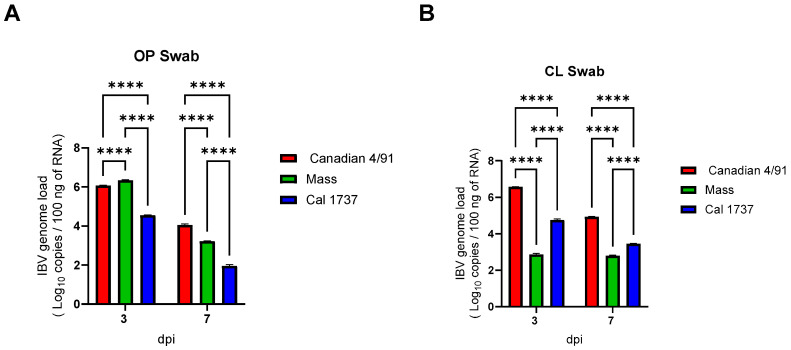
The mean IBV genome loads in 100 ng of extracted RNA from OP swabs (**A**) and CL swabs (**B**). Values are expressed as mean with SEM and were analyzed using two-way ANOVA followed by Tukey’s multiple comparison test. Significance: **** *p* < 0.0001.

Figure 1 illustrates the IBV genome loads in OP and CL swabs at 3 and 7 dpi with a significant difference between the three infected groups (*p* < 0.0001). A high IBV genome load in OP swabs was recorded in both 4/91- and Mass-infected groups (*p* < 0.0001), while a high CL excretion was detected in 4/91- and Cal 1737-infected groups (*p* < 0.0001).

**Figure 2:**
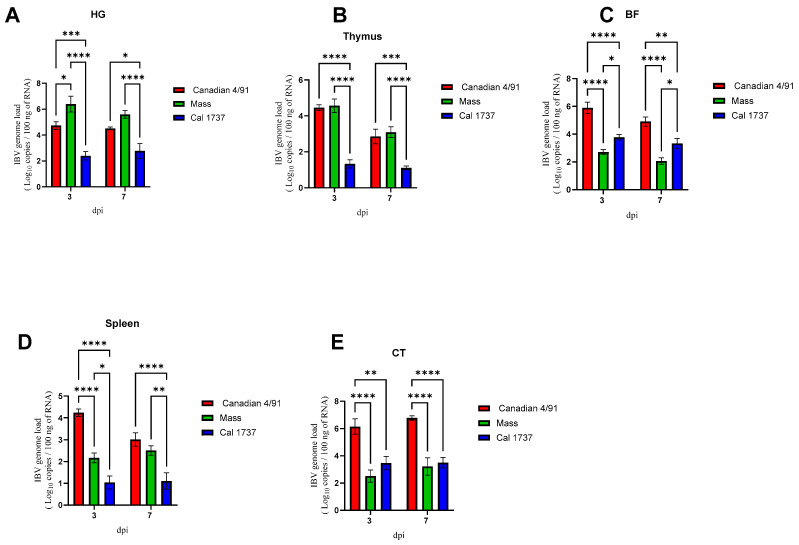
The mean IBV genome loads in 100 ng of extracted RNA from HG (**A**), thymus (**B**), BF (**C**), spleen (**D**), and CT (**E**) at 3 and 7 dpi of infected birds are shown. Values are expressed as mean with SEM and were analyzed using two-way ANOVA followed by Tukey’s multiple comparisons. Significance: * *p* < 0.05, ** *p* < 0.01, *** *p* < 0.001, **** *p* < 0.0001.

Dissemination of all the experimental IBV genotypes to the HG, thymus, BF, spleen, and CT tissues was seen from 3 dpi onwards (Figure 2). In the HG (Figure 2A), the highest viral genome load was observed in the Mass IBV-infected group at 3 dpi and 7 dpi (*p* > 0.05) compared to Cal 1737 IBV infected group. In the thymus, there was a significant elevation in the viral loads in both Canadian 4/91 and Mass IBV infected groups (Figure 2B) compared to Cal 1737 IBV-infected group at 3 (*p* > 0.0001) and 7 dpi (*p* > 0.001). Significant differences in viral loads were recorded among the three genotypes in BF at 3 and 7 dpi. Among them, the highest load was recorded in Canadian 4/91 IBV-infected group (Figure 2C). In the spleen, the highest genome load was detected in Canadian 4/91 IBV-infected group at 3 dpi., while at 7 dpi, there was no significant difference recorded between Canadian 4/91 and Mass IBV-infected groups (Figure 2D). The highest viral genome load was observed in the CT of Canadian 4/91 IBV-infected group (*p* < 0.01) in the absence of any significant difference between the viral genome loads of the Mass and Cal 1737 IBV-infected groups at both 3 and 7 dpi (Figure 2E).

### 3.2. Histopathological Examination

The control uninfected group revealed a normal histological architecture of the HG, BF, spleen, thymus, and CT. No histopathological lesions were observed in the spleen and thymus of all infected groups. 

**Figure 3:**
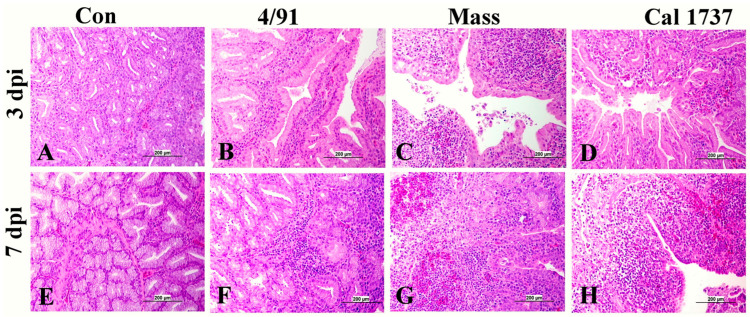
Representative histopathology photomicrograph of HG. (**A**,**E**) The mock control group shows normal histological structure. (**B**,**F**) Canadian 4/91 IBV-infected group shows mild inflammatory cell aggregation between the glands. (**C**,**G**) Mass IBV-infected group shows severe degeneration and necrosis of glands and hemorrhage with massive mononuclear inflammatory cell infiltration, mainly lymphocytes. (**D**) Cal 1737 IBV-infected group shows mild inflammation. (**H**) Cal 1737 IBV-infected group shows severe degeneration and necrosis of glands, hemorrhage, edema, and massive mononuclear inflammatory cell infiltration, mainly lymphocytes. The scale = 200 µm.

The HG was the immune organ affected the most. The nature of the lesions was mild in the Canadian 4/91-infected group when compared to the Mass and Cal 1737 IBV-infected groups. Figure 3 summarizes the HG lesions in all groups. The Canadian 4/91 IBV-infected group showed a significant increase in the score compared to the mock-infected group at 7 dpi (*p* < 0.0001). In the Canadian 4/91-infected group, HG histopathological changes were mild-to-moderate lesions in the form of inflammatory cell infiltration in lobules and interlobular septa with mild hyperplasia and necrosis of the secretory gland at 3 and 7 dpi (Figure 3B,F). The Mass IBV-infected group revealed moderate-to-severe lesions at both 3 and 7 dpi. The lesions were glandular hemorrhage, massive glandular epithelium necrosis, and intra- and interlobular edema with inflammatory cell infiltration mainly with heterophils, macrophages, and lymphocytes (Figure 3C,G). The Cal 1737 IBV-infected group revealed mild lesions at 3 dpi with massive and destructive lesions at 7 dpi (Figure 3D,H). The lesions had the same nature as Mass IBV-infected group.

**Figure 4:**
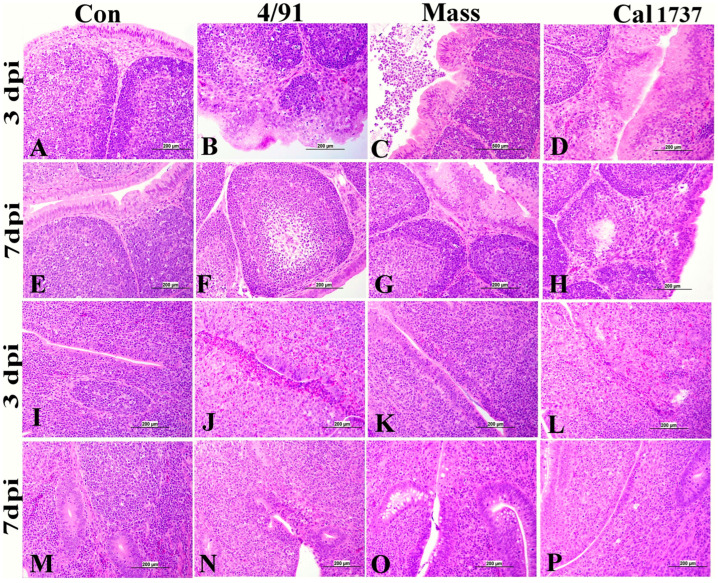
(**A**–**H**) Representative histopathology photomicrograph of BF. (**I**–**P**) Representative histopathology photomicrograph of CT. (**A**,**E**) Control group showing normal histological architecture. (**B**) Canadian 4/91 IBV-infected group shows severe lining epithelial hyperplasia and squamous cell metaplasia with degeneration and necrosis of most cells. (**C**) Mass IBV-infected group shows moderate hyperplasia of lining epithelium with accumulation of cellular debris and inflammatory cells in the lumen. (**D**) Cal 1737 IBV-infected group shows severe hyperplasia and squamous cell metaplasia of epithelial lining with apoptosis and necrosis of some cells. (**F**) Canadian 4/91 IBV-infected group shows moderate lymphoid depletion with expansion of interfollicular area with mononuclear inflammatory cells. (**G**) Mass IBV-infected group shows marked mild lymphoid follicle depletion and severe hyperplasia and squamous cell metaplasia of lining epithelial cells. (**H**) Cal 1737 IBV-infected group shows marked lymphoid follicle depletion, expansion of sub-epithelial tissue with inflammatory cells, and hyperplasia, degeneration, and necrosis of epithelial lining. (**I**,**M**) Control group shows normal histological picture of lympho-epithelium, subepithelial, and germinal center. (**J**) Canadian 4/91 IBV-infected group shows severe epithelial attenuation with epithelial and subepithelial inflammatory cell infiltration mainly with heterophils, lymphocytes, macrophages, and foamy macrophages. (**K**) Mass IBV-infected group shows mild epithelial and subepithelial mononuclear inflammatory cell aggregation. (**L**) Cal 1737 IBV-infected group shows severe epithelial lining necrosis with epithelial and subepithelial inflammatory cell infiltration mainly with heterophils, and mononuclear inflammatory cells with patchy areas of lymphoid necrosis. (**N**) Canadian 4/91 IBV-infected group shows moderate degeneration and necrosis of lympho-epithelium and mononuclear inflammatory cell aggregation. (**O**) Mass IBV-infected group shows moderate epithelial and mucus cell hyperplasia of lining epithelium with accumulation of cellular debris and inflammatory cells in the lumen. (**P**) Cal 1737 IBV-infected group shows moderate lymphoepithelial degeneration and necrosis with subepithelial lymphoid cell necrosis. The scale = 200 µm.

A significant difference in BF lesion score was recorded among Canadian 4/91, Mass, and Cal 1737 IBV-infected groups. Cal 1737 IBV-infected group showed the highest BF lesion score. Moderate-to-severe plical epithelial lining hyperplasia and squamous cell metaplasia with degeneration and necrosis of some cells and ballooning of mucus cells were the lesions observed in the BF of all infected groups (Figure 4A–H). In addition to this, subepithelial inflammatory cell aggregation mostly with heterophils, macrophages, and lymphocytes was also observed. Furthermore, lymphoid depletion was observed in lymphoid follicles.

**Figure 5:**
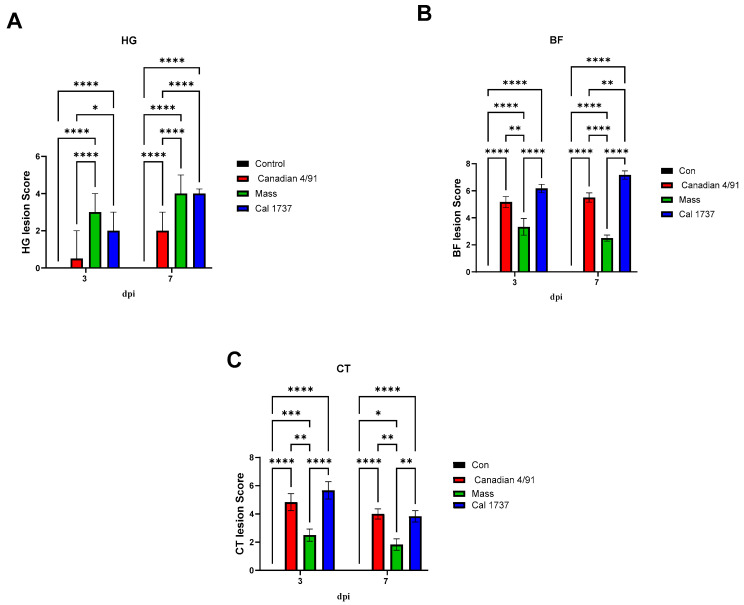
The lesions scores in lymphoid tissues in IBV-infected and mock control groups. (**A**) HG, (**B**) BF, (**C**) CT lesion scores. Values expressed as median with interquartile range and were analyzed using two-way ANOVA followed by Tukey’s multiple comparisons. Significance: * *p* < 0.05, ** *p* < 0.01, *** *p* < 0.001, **** *p* < 0.0001.

Figure 5 summarizes the CT lesion score in all IBV-infected groups. A significant difference in the CT score was observed among Canadian 4/91, Cal 1737, and Mass IBV-infected groups. CTs of all infected groups revealed moderate-to-severe degeneration and necrosis of their lympho-epithelium with mild-to-moderate subepithelial inflammatory cell infiltration especially with heterophils, foam macrophages, and mononuclear cells (Figure 4J–P). Apoptosis and lymphoid necrosis were observed in germinal centers and interfollicular areas. Canadian 4/91 and Cal 1737 IBV-infected groups revealed moderate-to-severe lesions compared to Mass IBV-infected group (Figure 4J–P).

### 3.3. Immunohistochemical Staining of IBV Nuclear Protein

The immune organs of the mock control group did not show any immune-positive staining of the IBV nuclear antigen. In contrast, the three infected groups exhibited positive IBV intra-cytoplasmic immune staining in the epithelial cells and macrophages of HG, BF, and CT tissues. Table 2 summarizes the expression of IBV nuclear protein expression in various organs sampled from different experimental groups. Cells with intra-cytoplasmic, brown fine-to-coarse crumbs were considered IBV immune-positive cells. The number of immune-positive tissues was counted out of six (total number of animals per group).

**Figure 6:**
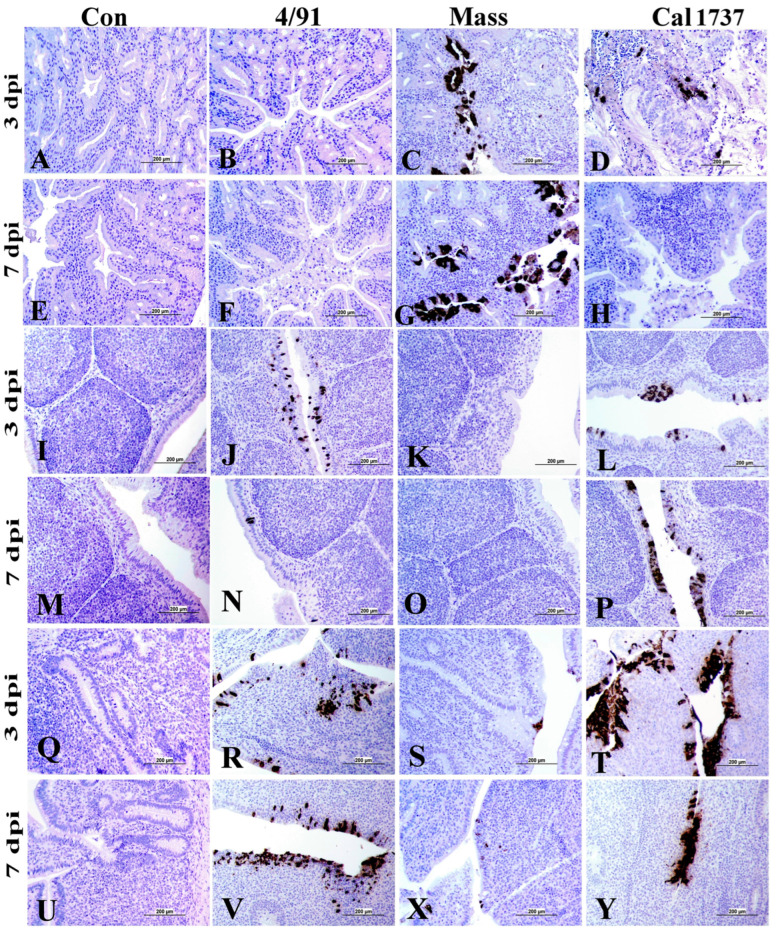
Immunohistochemical staining of IBV antigen in HG, BF, and CT. (**A**–**H**) HG; (**I**–**P**) BF; (**Q**–**V,X,Y**) CT. The scale = 200 µm. Cells with intra-cytoplasmic, brown fine-to-coarse crumbs were considered IBV immune-positive cells.

No IBV nuclear protein expression was recorded in the spleens of any IBV-infected groups. The HG of Mass and Cal 1737 IBV-infected groups showed positive IBV nuclear protein in the epithelial lining glands and macrophages (Figure 6C,D,G). In the BF, the IBV antigen expression was noticed in the columnar epithelium lining the plicae and in the macrophages that were detected in the subepithelial area (Figure 6J,L,N,P). Concerning the CT, positive staining was detected in the lympho-epithelium and underlying macrophages of CT of the IBV-infected groups (Figure 6R–T,V,X,Y).

### 3.4. Immunohistochemical Staining of B (BU-1+) and T (CD + 3) Cells

**Figure 7:**
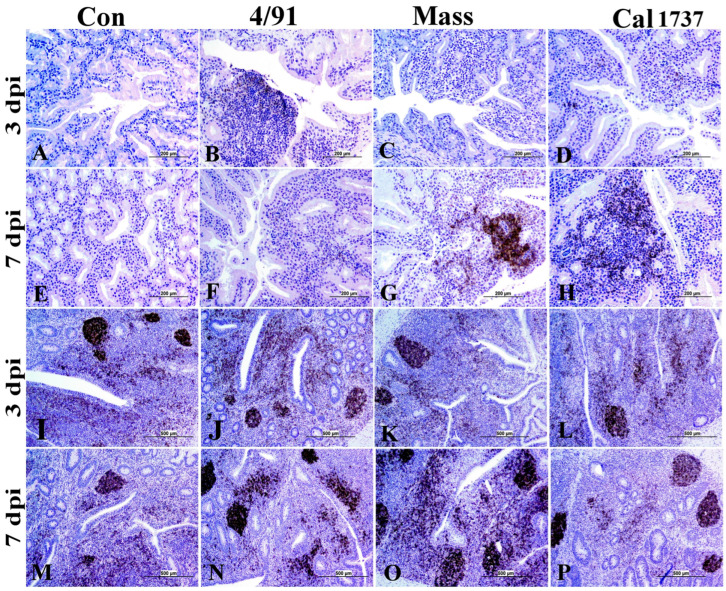
Representative photomicrograph of BU-1 immune-positive cells. (**A**–**H**) HG and (**I**–**P**) CT. The scale = 500 µm.

**Figure 8:**
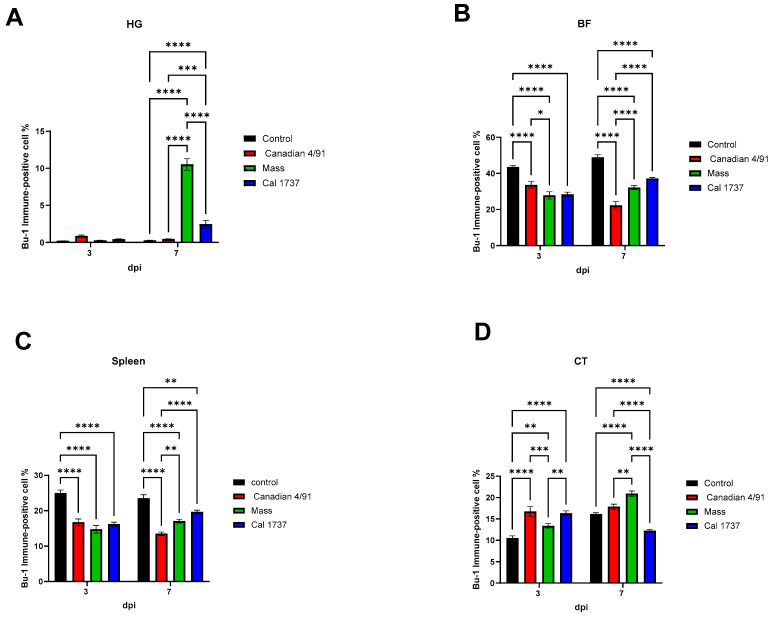
BU-1 immune-positive cell % in observed lymphoid tissues in IBV-infected and mock control groups. (**A**) HG, (**B**) BF, (**C**) spleen, and (**D**) CT. Values expressed as mean + SE and were analyzed using two-way ANOVA followed by Tukey’s multiple comparisons. Significance: * *p* < 0.05, ** *p* < 0.01, *** *p* < 0.001, **** *p* < 0.0001.

Figure 7 and Figure 8 illustrate the BU-1 immune-positive cell % in the different tissues originating from IBV-infected and mock control groups. No significant difference between the mock control group and IBV-infected groups was observed in the HG at 3 dpi. Only the HGs of the Mass and Cal 1737 IBV-infected groups showed significant differences in the BU-1 immune-positive cell % when compared to the normal control group at 7 dpi (*p* < 0.0001). The Mass group showed the highest BU-1 expression among all groups at 7 dpi (Figure 8A, *p* < 0.0001). In BF, all IBV-infected groups showed a significant reduction in the BU-1 immune-positive cell % compared to the normal control group at both 3 and 7 dpi (*p* < 0.0001). The Mass and Cal1737 IBV-infected groups revealed the lowest percentage of BU-1 immune-positive cells at 3 dpi (Figure 8B, *p* < 0.0001). However, at 7 dpi, the Canadian 4/91 IBV-infected group showed the lowest BU-1 immune-positive cell % (*p* < 0.0001). In the spleen, all IBV-infected groups exhibited a significant reduction in BU-1 expression compared to the mock control group (*p* < 0.01) at both 3 and 7 dpi (Figure 8C). In contrast, the CTs of Canadian 4/91, Mass, and Cal 1737 groups showed a significant elevation in the BU-1 immune-positive cell % compared to the mock control group (*p* < 0.01) at both 3 and 7 dpi (Figure 8D).

**Figure 9:**
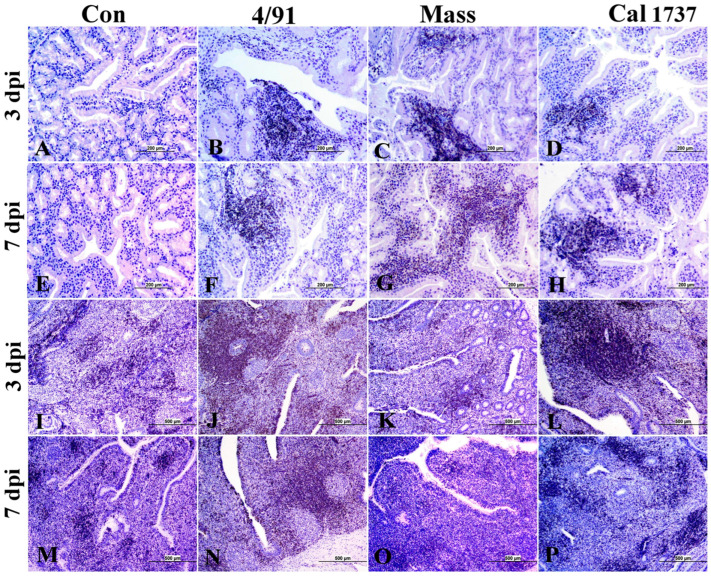
Representative photomicrographs of CD3 immune-positive cells. (**A**–**H**) HG and (**I**–**P**) CT. The scale = 500 µm.

**Figure 10:**
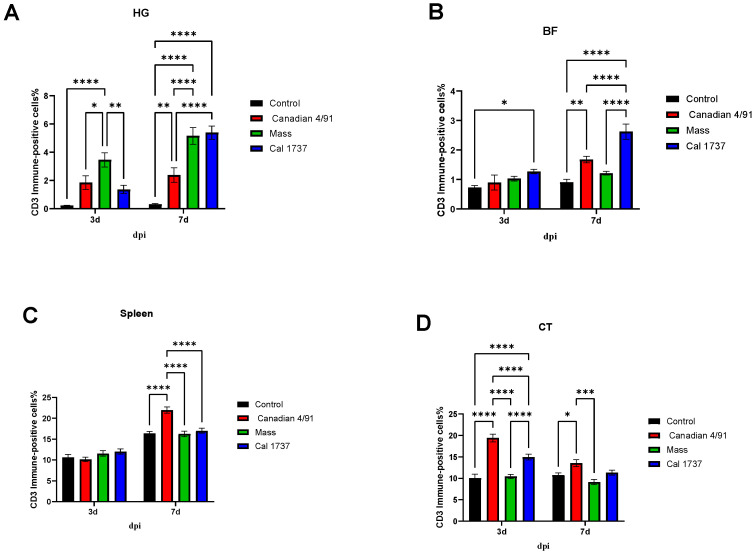
CD3 immune-positive cell % in observed lymphoid tissues in IBV-infected and mock control groups. (**A**) HG, (**B**) BF, (**C**) spleen, and (**D**) CT. The values are expressed as mean + SE and were analyzed using two-way ANOVA followed by Tukey’s multiple comparisons. Significance: * *p* < 0.05, ** *p* < 0.01, *** *p* < 0.001, **** *p* < 0.0001.

Figure 9 and Figure 10 illustrate the CD3 immune-positive cell % in the observed lymphoid tissues. In the HG at 3 dpi, Mass IBV-infected group was the only group showed a significant increase in the CD3 immune-positive cell % when compared with the mock control group (*p* < 0.0001). But at 7 dpi, the three IBV-infected groups revealed significant elevations in the CD3 immune-positive cell % compared to the mock control group (*p* < 0.01, *p* < 0.0001). In the BF, no significant difference was recorded between the IBV-infected groups and the mock control group at 3 and 7 dpi except the Cal 1737 IBV-infected group at both time points and the Canadian 4/91 IBV-infected group at 7 dpi. There was not any significant difference in the CD3 immune-positive cell % in the spleens of the mock control group and infected groups at both time points, except the Canadian 4/91 IBV-infected group showed a significant elevation at 7 dpi (*p* < 0.0001). The Canadian 4/91 IBV-infected group was the only group that showed a significant elevation in the CD3 immune-positive cell % in the CT at both time points (*p* < 0.0001, *p* < 0.05, respectively), while the Cal 1737 IBV-infected group showed this significant difference at 3 dpi only (*p* < 0.0001).

## 4. Discussion

IBV infection remains a substantial poultry health problem, in spite of employing intensive vaccination programs [50]. IBV can infect all chickens in all age categories and replicate in a variety of tissues, leading to significant pathology. It is known that the IBV genotypes differ in their virulence, pathogenicity, and tropism [51]. Although a myriad of previous studies has focused on the pathogenesis of various IBV genotypes on the respiratory, urinary, and reproductive tracts, studies are limited on the impact of IBV on lymphoid organs. Therefore, in our study, we investigated the impact of IBV strains on primary and secondary lymphoid organs. Our observations revealed that the Canadian 4/91, Mass, and Cal 1737 IBV genotypes led to histological changes following replication in lymphoid organs such as the HG, BF, and CT with the consequence of a reduction in BU-1-positive B cells in the BF and spleen and an elevation in CD3+ T cells in the HG and CT.

The ocular, nasal, and oral mucosal membranes are the main ports of entry of IBV [52]. The chicken HG is related to mucosal immunity that protects the host against pathogen invasion [34]. Several previous studies have demonstrated the role of plasma cells in the HG in the production of antibodies in an antigen-specific manner [34,53,54,55,56]. After IBV infection, HGs were severely enlarged due to inflammation and immunoreactive cellular responses [52]. In the present study, we observed that the tested IBV genotypes could induce a range of lesions. The observed replication of IBV in the HG epithelial cells of Mass and Cal 1737 IBV-infected chickens agrees with previous studies [34,57]. On the other hand, no IBV antigen was observed in HG sections of Canadian 4/91-infected chickens at the observed time points with a considerable amount of IBV genome quantified. IBV genome load represents both the active and inactive form of the virus, while the detection of IBV antigens indicates active viral replication [58]. In the case of the Canadian 4/91 IBV-infected group, the negative IBV antigen might be due to the rapid control of virus replication, and this was associated with the presence of less severe microscopic lesions in this group. Overall, we confirmed that the three different IBV genotypes had a varying affinity to replicate and induce severe lesions in HG and that viral replication in the HG is dependent on the infecting genotype of IBV as seen by the severity of histological changes.

In our study, irrespective of the IBV genotype used, neither histopathological lesions nor positive IBV nuclear protein expression was detected in the spleen following IBV infection. Like many previous studies, IBV genome load was detected in the thymus and spleen of infected birds [17,31,32,35]. However, a low IBV genome load was quantified, suggesting that IBV infects the spleen, but clearance is quicker than that observed for the HG, thymus, BF, and CT. It might be also explained by an absence or limited number of epithelial cells in the spleen and thymus, respectively [59], which are the main viral target cells [60]

Our observation of IBV-induced BF lesions agrees with the findings that have been observed previously [31,32,33,35] and confirmed the virus replication in the BF. Our results indicated that there were clear differences in IBV genome load, IBV replication, and BF lesions among the three IBV genotypes. The absence of viral antigen in the BF epithelial cells of the Mass IBV-infected group was not surprising, as the bursal lesions in these chicks were mild with a very low detected IBV genome load. In a previous study, it was observed that the Mass IBV strain had limited viral replication in the bursal epithelium lining [33], whilst the Canadian 4/91 and Cal 1737 IBV-infected groups showed significant elevations in IBV genome load and BF lesion scores with marked viral replication in the plicae epithelium. These observations exhibited that the Canadian 4/91 and Cal 1737 IBV genotypes induce more severe lesions on the BF compared to those induced by the Mass IBV genotype, endorsing that each IBV strain has its own virulence and tissue tropism.

In this study, the 4/91 and Cal 1737 IBV-infected groups showed a significant elevation in viral genome load, nuclear protein expression, and lesion scores in the CT compared to the Mass IBV-infected group and these observations are in agreement with previous studies [28,41]. We observed that the three IBV strains analyzed replicate to different levels in the CT. The replication of the Mass strain is minimal as compared to the Canadian 4/91 and Cal 1737 strains. It has also been proposed in previous studies [28,41,61,62] that the CT is the site of IBV persistence.

During IBV infection in chickens, cell-mediated immunity is one of the indispensable immunoregulatory mechanisms for viral clearance, decline of infection, and virus shedding, in addition to vaccine response [40,41]. IBV replication in immune organs, particularly in secondary lymphoid organs, is expected to stimulate a vigorous immune response in these organs [34]. Recruitment of lymphocytes in the visceral organs following IBV infection was previously recorded in many studies [21,63]. In the current study, B- and T-lymphocyte responses in all immune organs showed a clear difference among strains analyzed, Mass, 4/91, and Cal 1737, at 7 dpi.

There was a significant decline in BU-1 expression in the BF and spleen of the 4/91, Mass, and Cal 1737 IBV-infected groups compared to the mock control group. This observation of B-cell depletion following IBV infection in the BF could be explained by previous studies [64]. Farsang and colleagues recorded that the indirect follicular destruction in BF during IBV infection is possible via the impaired development of the corticomedullary arterioles in this tissue [64]. A similar explanation for B-cell depletion in the spleen is not available, and further studies are required to investigate if IBV directly targets B cells in the BF and spleen. The HG and CT of the IBV-infected groups showed a significant elevation in the number of B cells. The higher IBV replication in these lymphoid organs is expected to stimulate a vigorous immune response in these organs in the form of massive lymphocytic infiltration. Our results are in agreement with the work of others that reported the number of lymphocytes in the HGs and CTs of IBV-infected chickens was four times more than uninfected controls, and the increased lymphocytes included B cells [28,65]. A marked decline in B cells in the BF and spleen of all experimentally infected groups suggests the immunosuppressive effect of the 4/91, Mass, and Cal 1737 genotypes of IBV.

Unlike B-cell depletion, we did not observe any decrease in CD3+ T cells in any of the observed lymphoid organs following IBV infection. In fact, the number of CD3+ T cells was either not changed or increased significantly following IBV infection. The increase in T cell numbers may indicate a possible cell-mediated immune response generated against IBV replication in these lymphoid organs [28,65] or a lack of IBV tropism towards CD3+ T cells. In either case, further studies are required.

## 5. Conclusions

In conclusion, the tissue tropism, pathogenicity, replication, and induced cell-mediated immune response in the lymphoid organs of the Canadian 4/91, Mass, and Cal 1737 strains of IBV differ from each other. The histopathological assessment and immunohistochemical staining verified that IBV antigens were present in the HG, BF, and CT, resulting in notable tissue damage and a decrease in B-cell numbers in the spleen and BF in all infected groups, which may potentially lead to immunosuppression. Further studies are needed to understand the functional consequences of IBV-induced pathology and B-cell depletion in lymphoid organs.

## Figures and Tables

**Table 1:** The lesion scoring criteria for immune organs of infected birds.

Organ	Scoring Criteria
Harderian gland	Secretory gland necrosis Interlobular edema Inflammatory cell infiltration Hemorrhage
BF	Lymphoid follicle depletion Epithelial lining hyperplasia with squamous cell metaplasia Epithelial lining degeneration and necrosis
Thymus	Depletion of the cortical lymphocyte density or relative thickness to the medulla Thymus hemorrhage
Spleen	Hyperplasia or hypertrophy in the ellipsoids Lymphoid apoptosis or necrosis Sinusoidal congestion
CT	Lymphoepithelial degeneration and necrosis Subepithelial zone inflammatory cell infiltration Lymphoid apoptosis and necrosis in germinal centers

**Table 2:** Expression of IBV nuclear protein expression in observed lymphoid tissues.

Lymphoid Tissue	HG	Thymus	Spleen	BF	CT
Control—3 and 7 dpi	0/6	0/6	0/6	0/6	0/6
4/91—3 dpi	0/6	3/6	0/6	5/6	6/6
4/91—7 dpi	0/6	4/6	0/6	4/6	6/6
Mass—3 dpi	3/6	4/6	0/6	0/6	3/6
Mass—7 dpi	6/6	5/6	0/6	0/6	1/6
Cal 1737—3 dpi	2/6	3/6	0/6	5/6	6/6
Cal 1737—7 dpi	0/6	3/6	0/6	3/6	5/6

## Data Availability

The datasets used and/or analyzed within the frame of the study can be provided by the corresponding author upon reasonable request.
